# Urgent Endoscopic Biliary Procedures: “Run Like the Wind”?

**DOI:** 10.3390/jcm14031017

**Published:** 2025-02-05

**Authors:** Francesca Lodato, Stefano Landi, Marco Bassi, Stefania Ghersi, Vincenzo Cennamo

**Affiliations:** Department of Gastroenterology and Interventional Endoscopy, AUSL Bologna Bellaria, Maggiore Hospital, 40133 Bologna, Italy; stefano.landi@ausl.bo.it (S.L.); marco.bassi@ausl.bo.it (M.B.); stefania.ghersi@ausl.bo.it (S.G.); vincenzo.cennamo@ausl.bo.it (V.C.)

**Keywords:** biliary endoscopy, emergency, organization

## Abstract

Emergency endoscopy is an activity that must be guaranteed 7 days a week and 24 h a day. The pathologies of endoscopic interest that require emergency intervention are mainly hemorrhages of the upper digestive tract, the removal of foreign bodies, and the ingestion of caustics. The emergency endoscopist must therefore be experienced in the management of these pathologies. Nowadays, however, we know that even some biliary tract pathologies must be managed within a variable period between 12 and 72 h, in particular acute cholangitis (Ach), acute biliary pancreatitis (ABP), biliary duct leaks (BDLs), and acute cholecystitis (AC). If, on one hand, there is little awareness among doctors about which pathologies of the biliary tract really deserve urgent treatment, on the other, the international guidelines, although not uniformly, have acquired the results of the studies and have clarified that only severe Ach should be treated within 12 h; in other cases, endoscopic treatment can be delayed up to 72 h according to the specific condition. This obviously has a significant organizational implication, as not all endoscopists have training in biliary tract endoscopy, and guaranteeing the availability of a biliary endoscopist 24/7 may be incompatible with respecting the working hours of individual professionals. This review aims to evaluate which pathologies of the biliary tract really require an endoscopic approach in emergency or urgency and the organizational consequences that this can determine. Based on the guidelines, we can conclude that a daytime availability for urgent biliary tract procedures 7 days a week should be provided for the management of severe ACh. Patients with ABP, AC unfit for surgery, and not responsive to medical therapy or BDLs can be treated over a longer period, allowing its scheduling on the first available day of the week.

## 1. Introduction

The organization of digestive endoscopy units provides for an on-call endoscopist 24 h a day. This figure is needed to manage urgent and emergent conditions such as digestive hemorrhages, caustic ingestion, or the ingestion of foreign bodies. In these cases, the promptness of the procedure plays a role in the patient’s prognosis depending on the clinical situation [1,2,3,4]. Therefore, the emergency endoscopist must be trained in the management of endoluminal urgencies.

Urgent endoscopic procedures are also often required for some biliopancreatic diseases. Training in biliopancreatic endoscopy is specific, and not all endoscopists can perform such procedures. In addition, indications for performing emergency or urgent procedures in biliopancreatic diseases are uncertain, and it is unclear if promptness of the procedure has a prognostic impact.

This review focuses on biliopancreatic diseases that may benefit from performing endoscopic procedures in emergency/urgent setting, namely acute cholangitis (ACh), acute biliary pancreatitis (ABP), biliary duct leaks (BDLs), and acute cholecystitis (AC); moreover, we discuss the practical–organizational impact that the proper management of biliary emergencies can have.

## 2. Acute Cholangitis (Ach)

ACh is a common clinical complication of biliary obstruction associated with significant morbidity and mortality. The mortality rate ranges from 10% to 40%, depending on disease severity [5]. Thus, severity assessment is a cornerstone for ACh management.

The 2013 revision of the Tokyo Guidelines [6], confirmed in 2018 [7], divides ACh into severe, moderate, and mild, considering severe ACh associated with other organ systemic dysfunctions (Table 1).

Most patients with ACh have mild-to-moderate disease responding to antibiotics therapy, which is a cornerstone in these cases [9,10]; but in 15–30% of cases, ACh is severe, requiring prompt biliary decompression; therefore, a correct classification of cholangitis severity is fundamental to selecting the appropriate treatment.

Endoscopic retrograde cholangiopancreatography (ERCP) remains the procedure of choice for biliary drainage, and its superiority over surgical procedures in terms of mortality and morbidity has already been established in many studies [10].

A further advantage of ERCP is the possibility of performing a biliary culture; this may be of particular importance given the increasing occurrence of resistance to third-generation cephalosporins, the most used antibiotics in patients with acute cholangitis [11,12].

In case of ERCP failure or unfavourable anatomy, percutaneous transhepatic biliary drainage (PTBD) or, more recently, endoscopic ultrasound-guided biliary drainage (EUS-BD) is an alternative approach to ERCP recommended by international guidelines [5,13].

The questions are as follows: When should ERCP be performed? How urgent is the procedure? In this topic, the literature is not in complete consensus.

The current Tokyo guidelines recommend biliary drainage in severe ACh “as soon as possible after patients’ conditions allows”; in cases of mild and moderate ACh not responding to medical treatment, early biliary drainage is recommended but no specific timing for procedure is given [7].

Most studies chose to compare procedures when performed before or after 48 h mostly because they address the workforce and financial concerns of weekend procedures [14].

No randomized trials have compared ERCP before 48 h versus >48 h but a recent systematic review and meta-analysis by Iqbal et al. [14] defined “emergent ERCP” as a procedure performed within 48 h and found that in severe ACh, this procedural timeframe was associated with significantly lower in-hospital mortality and a shorter duration of hospital stay. Emergent ERCP was not only beneficial in patients with severe ACh but it was associated with better outcomes in patients with mild to moderate ACh in terms of 30-day mortality and organ failure. The meta-analysis included studies with patients who underwent ERCP within 24 or 48 h, but it was impossible to evaluate any mortality difference in patients with ACh who underwent ERCP within 24 h and patients who underwent ERCP between 24 and 48 h, because the mortality data for patients who underwent ERCP between 24 and 48 h was inconsistently reported in the studies [15,16,17,18,19,20,21,22,23,24,25]. Accordingly, the American Society of Gastrointestinal Endoscopy (ASGE) guidelines on the management of acute cholangitis recommend the performance of ERCP within 48 h for biliary decompression [5].

Nevertheless, the opportunity of performing ERCP “as soon as possible” as suggested by Tokyo Guidelines is also supported by a 2016 international study [26] including 260 patients with septic shock; the authors found that biliary drainage performed more than 12 h after the onset of shock was associated with higher in-hospital mortality (OR 3.4, 95% CI 1.12–10.31).

As a result, the European Society of Gastrointestinal Endoscopy (ESGE) Guidelines [13] are more detailed in the definition of timing for biliary drainage and recommends performing ERCP as soon as possible in severe ACh and within 12 h for patients with septic shock. When ACh is moderate, it is suggested to be performed within 48–72 h. For mild cases, the procedure could be elective.

Another important point is whether the timing of biliary drainage is equally important in malignant diseases. Malignant biliary obstruction (MBO), such as pancreatic cancer, cholangiocarcinoma, or metastatic cancer, is observed in 10–30% of cholangitis [22,23,27].

Neoplastic patients are certainly more complex than those suffering from lithiasis; their performance status is compromised by malnutrition related to concomitant chemotherapies and neoplastic cachexia [28,29,30]. Overall, they have a worse prognosis with a higher risk of readmission within 30 days [31] and, when ACh is present, it is associated with higher mortality and morbidity [32].

Moreover, when a biliary stricture is present, an additional endoscopic treatment is required to achieve an effective biliary decompression, such as stent placement, which could be more challenging in these patients due to anatomical alterations related to underlying disease [33,34].

A retrospective study [35] including 421 patients with ACh and distal MBO evaluated three time frames for ERCP: the authors chose to define “urgent” as the procedures performed within 24 h from admission; “early” as the procedures performed between 24 and 48 h; and “late” as the procedures performed after 48 h. ERCP performed by 24 h was significantly related to lower 30-day mortality (urgent 2.2%, early 4.3%, late 13.5%; *p* < 0.001) and 180-day mortality (39.4%, 44.8%, 60.8%; *p* = 0.006); these results were confirmed in subgroup analysis both for 30- and 180-day mortality, for patients with primary MBO and with moderate-to-severe cholangitis.

These data should support increased attention and urgency in patients with MBO, who may have a clinical presentation less conspicuous of ACh than patients with lithiasis. Nevertheless, a retrospective study comparing the waiting time for ERCP in patients with ACh and MBO compared to those with ACh and lithiasis showed that it is significantly longer in patients with MBO [36]. In this study, MBO-related ACh had a significantly higher 30-day mortality than lithiasis-related Ach, with time to ERCP, but not MBO itself, being an independent factor associated with 30 d mortality in the multivariate analysis. Other relevant findings were the significantly higher ICU admission and the 30-day readmission rates in patients with MBO. Therefore, we can conclude that more attention should be paid to neoplastic patients with MBO, offering these patients urgent procedures that guarantee better outcomes.

## 3. Acute Biliary Pancreatitis (ABP)

Acute pancreatitis (AP) is a frequent condition that can have different presentations with a benign outcome in most cases. In 20% of patients, however, the presentation is severe and is associated with high mortality and morbidity because patients may develop cholangitis, organ failure, and other severe complications [37].

The main etiology of AP is biliary, due to obstruction of the biliary tract by stones. This may result in obstruction and bile reflux into the pancreatic duct, which can trigger the processes of self-digestion by pancreatic enzymes. Clinical and experimental studies have shown that the severity of pancreatitis depends on the duration of biliary obstruction [38,39], with 80% of severe pancreatitis reported when an obstruction persists more than 48 h. Therefore, the need to drain the biliary tract by endoscopic decompression, mainly using ERCP with or without sphincterotomy, is mandatory.

The timing of the procedure, however, is debated; while it may be intuitive that the sooner the cause is removed, the sooner the pancreatitis is resolved, this is not universally true nor is it substantiated by clear evidence.

The first trial on the use of urgent ERCP in ABP was in 1988 [40]; the study evaluated the outcome of patients hospitalized for ABP and showed a significant reduction in complications (fluid collection rate and organ failure rate) and mortality in patients with severe ABP treated by ERCP within 72 h from ABP onset, compared with those treated conservatively. In 1993, Fan et al. [41] confirmed these results in a group of patients admitted with AP with various etiologies, mainly biliary. In this study, patients were randomly assigned to the early ERCP or conservative group even though gallstone diagnosis was not confirmed. Early ERCP was established in 24 h.

Later, other studies confirmed these results, albeit with some differences especially on the definition of the time of urgency (24 vs. 72 h) [42,43,44,45,46,47], as summarized in Table 2.

Regarding possible complications of the procedure, the advantage of biliary clearance remains significant: the main post-procedural complication was represented by bleeding, which, however, does not appear to increase mortality in a large cohort study comparing mortality in patients that underwent early or late ERCP with ABP (1.1 vs. 0.57, OR 2.08) [48].

**Table 2:** Main studies comparing early ERCP (at different timepoints) with conservative treatment for AP with cholangitis.

Author	Year	Patients (ERCP/Control)	Timing (h)	Mortality (%)	Morbidity (%)	Pancreatic Fluid Collection (%)	Organ Failure/Sepsis (%)
Neoptolemos [40]	1988	59/62	<72	1.7/8.1	22/62.9	11.9/21.0	10.2/37.1
Fan [41]	1993	97/98	<24	5.2/9.4	20.6/38.8	10.3/12.2	10.3/26.5
Nowak [47]	1998	178/102	<24	2.2/12.8	16.9/38.2	-	-
Zhou [46]	2002	20/25	<24	0/0	5/16	5.0/16.0	-
Acosta [45]	2006	30/31	<48	0/0	3.3/25.8	3.3/22.6	0/3.2
Chen [44]	2010	21/32	<72	0/6.3	4.8/18.8	4.8/18.8	-
Zhou [43]	2011	50/55	<72	0/1.8	12/30.6	2.0/0	6.0/18.2
Yang [42]	2012	60/60	<72	1.7/10	10/26.7	1.7713.3	1.7/13.3

Modified from Mukai et al. [49].

Considering the above, it might seem clear, therefore, that ABP always needs a rapid ERCP with biliary drainage, even considering the greater technical and procedural difficulty observed in these patients [50].

On the other hand, if we think about the pathogenesis of pancreatitis, it is necessary to remember that, after stones migrate into the main bile duct and trigger pancreatitis, the same stone may pass spontaneously due to increased bile pressure in the duct, without causing cholangitis.

All studies described above have in common that they mostly included patients with cholangitis, jaundice, or cholestasis. ABP without cholangitis should be considered a different condition compared to ABP with cholangitis, and the efficacy of ERCP in this context has been evaluated in recent studies [51,52,53] and, as a result, no benefit has been shown from performing urgent biliary clearance, neither in terms of mortality nor major complications (Table 3).

A recent multicentre randomized trial on this issue published by Shepers et al. [51], which included only patients with predicted severe ABP without cholangitis, concluded that urgent ERCP with sphincterotomy did not reduce the composite endpoint of major complications or mortality, compared with conservative treatment. Their findings support a conservative strategy in patients with predicted severe ABP with an ERCP indicated only in patients with cholangitis or persistent cholestasis.

The same conclusions were proposed by an earlier Cochrane review in 2012 [54] that, pooling together seven RCTs including patients with and without severe pancreatitis, failed to show a significant benefit of early ERCP in terms of mortality (RR 0.74, 95% CI 0.18 to 3.03) and local and systemic complications as defined by the Atlanta Classification (RR 0.86, 95% CI 0.52 to 1.43; and RR 0.59, 95% CI 0.31 to 1.11, respectively), except for patients with cholangitis and cholestasis in whom early routine ERCP significantly reduced mortality (RR 0.20, 95% CI 0.06 to 0.68) and local and systemic complications as defined by the Atlanta Classification (RR 0.45, 95% CI 0.20 to 0.99; and RR 0.37, 95% CI 0.18 to 0.78, respectively).

Based on this evidence, ESGE recommends urgent ERCP in patients with ABP only in the presence of cholangitis or evidence of biliary obstruction [55].

As stated in ESGE guidelines, no study has been specifically designed to assess the timing of ERCP in biliary acute pancreatitis. The 2012 Cochrane systematic review did not show significant benefit when ERCP was performed as early as 24 h with respect to procedures delayed up to 72 h.

Considering this, the ESGE suggests performing ERCP within 24 h in cases of ABP with cholangitis, while delaying the procedure until 72 h in cases of biliary obstruction.

## 4. Biliary Duct Leak (BDL)

BDLs affects up to 1.5% of laparoscopic cholecystectomies [56,57,58,59,60,61]; it is moreover the most common surgical complication of liver transplantation [62,63,64], partial hepatectomy, and hepatic trauma [65].

ERCP with stenting, sphincterotomy, or a combination thereof is the first-line treatment for most BDLs, allowing the pressure gradient between the bile duct and the duodenum to be decreased; this creates a preferential transpapillary bile flow and allows the leak to heal [66,67]. The best approach to manage BDLs is not standardized, and current international guidelines recommend biliary decompression by either sphincterotomy alone or nasobiliary drain placement, with or without sphincterotomy (ASGE) [68] or biliary stent placement, only without sphincterotomy to avoid both short- and long-term adverse events (ESGEs) [69].

ERCP is effective in BDL treatment in up to 90% [70,71]; the remainder require additional treatment, with the location of the leak being reported as the main factor influencing the success of ERCP therapy [71]. In these cases, a second attempt could be performed with repeated stenting or the addition of a sphincterotomy if not previously performed. In refractory leaks, percutaneous transhepatic biliary drainage (PTBD) or surgical intervention should be proposed.

Some endoscopists and surgeons consider BDLs to be an emergency, whereas others treat patients on an elective basis [72].

No RCTs are available to evaluate the best timing for endoscopic treatment of biliary leaks. To the best of our knowledge, only three retrospective studies have studied the clinical impact of timing on ERCP outcome for the treatment of post-surgical BDL [73,74,75]. These studies divided patients into those exhibiting ERCP within 1 day, 2–3 days, and after 3 days from the onset of BDL. Studies are difficult to compare because of many differences: firstly, the BDL prevalence of post-cholecystectomy was 52%, 70.6%, and 100%, respectively; the rate of percutaneous abdominal drainage placement was as follows: not reported, 45%, and 9%, respectively. However, all three studies found that patients treated earlier have worst outcomes than those treated later, with lower overall adverse events after 3 days [75] and lower mortality when ERCP was performed between 48 and 72 h compared to those performed within 24 or after 72 h. These results could be explained with selection bias because it is likely that more severe patients underwent ERCP earlier while more stable cases were treated later with expectable better outcomes.

Nevertheless, in view of these data, there is no basis for considering BDLs as a matter of emergency and patients can mostly be managed within 3 days depending on the general clinical picture.

## 5. Acute Cholecystitis (AC)

AC is the most common complication of gallstone disease and the first presentation in up to 15% of cases [76,77].

According to the Tokyo guidelines, AC should be classified in three grades according to severity into mild, moderate, and severe [8], with grade 3 being associated with systemic dysfunction.

**Figure 1:**
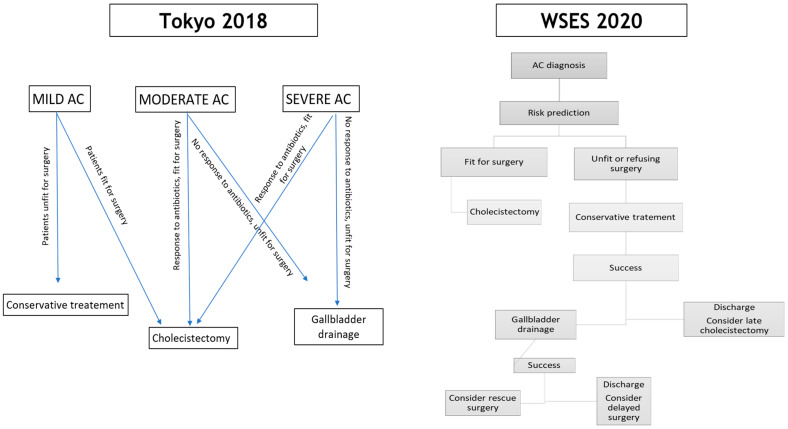
Flowchart of AC management according to Tokio and World Society of Emergency Surgery (WSES) Guidelines.

The definitive treatment of AC is cholecystectomy to be performed as soon as possible and within 7 days from admission, as stated by current guidelines [8,78], but in cases of patients unfit for surgery with severe AC not responding to antibiotics, non-surgical treatments (NSTs) should be proposed (Figure 1). The main factors predicting failure of NST in patients with absolute contraindication for surgery after 24 h of follow-up are age over 70 years, diabetes, tachycardia, and gallbladder empyema; after 48 h, predictors of failure are leucocytosis (>15,000 cell/mm^3^), fever, and age over 70 years. Therefore, in patients unfit for surgery, gallbladder drainage should be proposed and performed within 24–48 h [79] in case of non-response to medical treatment.

Among alternative approaches, AC could be treated by percutaneous transhepatic ultrasound/CT-guided biliary drainage (PT-GBD), transpapillary drainage by ERCP (TP-GBD), or EUS-guided transmural drainage (EUS-GBD).

PT-GBD is a feasible radiological procedure, with a rate of procedural success up to 86% [80,81]; however, mortality and morbidity is difficult to evaluate due to the significant heterogeneity of patients’ inclusion in different studies, ranging, respectively, from 4 to 50% for mortality and from 8 to 62% for morbidity [82]. Moreover, it is not a definitive treatment, since the gallbladder is not removed and possible recurrent biliary complications may occur [83,84]. A recent RCT comparing laparoscopic cholecystectomy and PT-GBD reported a significantly higher rate of major complications, reinterventions, and recurrent biliary disease in the PT-GBD group compared to surgery, even in high-risk patients [85].

TP-GBD can be performed with two different methods: endoscopic naso-gallbladder drainage and endoscopic gallbladder stenting. These transpapillary procedures are used to place a drainage tube in the gallbladder via the cystic duct with ERCP [86,87]. This approach is clinically effective but technically challenging as it needs selective cystic duct cannulation and stent placement, in addition to the complexity of a standard ERCP; thus, it should be reserved to patients with contraindications to other drainage approaches, like patients with ascites, severe coagulopathy, thrombocytopenia, or an anatomically inaccessible location or in patients undergoing ERCP for common bile duct obstruction [88]. This approach could reasonably be performed in patients undergoing ERCP for biliary obstruction.

EUS-GBD is a relatively new technique; it is based on the creation of a fistula between the stomach or the duodenal bulb and the gallbladder using a stent, fixing the gallbladder wall directly to the intestinal lumen [89,90,91]. In the beginning, different devices were adapted from ERCP or transmural drainage, like self-expanding metal stents (SEMSs) or double pig-tail plastic stents (DPSs). Those devices have several problems when used for EUS-GBD, being designed for other purposes. SEMSs with radial expansion may be traumatic to visceral walls and might migrate due to the lack of anti-migratory systems; DPSs have a higher risk of leakage due to the lack of sealing of the fistula [91]. More recently, a novel technique for EUS-guided positioning of a lumen-apposing metal stent (LAMS) was implemented. LAMSs are self-expanding metal stents with a dedicated design for transmural drainage; they have flanges to ensure lumen-to-lumen apposition, anti-migratory properties, and sealing of the fresh transmural tract, minimizing the risk of leakage [92].

The performance of EUS-GBD and PT-GBD has been evaluated in only one RCT [93]; both procedures had comparable technical and clinical success rates (97.4% vs. 100%, *p* = 0.494 and 92.3% vs. 92.5%, *p* = 1, respectively) with comparable 30-day mortality (7.7% vs. 10%, *p* = 1). Nevertheless, EUS-GBD was associated with significantly lower (25.6% vs. 77.5%, *p* < 0.001) 30-day adverse events (12.8% vs. 47.5%, *p* = 0.010), re-interventions after 30 days (2.6% vs. 30%, *p* = 0.001), readmissions (15.4% vs. 50%, *p* = 0.002), and recurrent cholecystitis (2.6% vs. 20%, *p* = 0.029). PT-GBD was also related to an increased risk of cholecystitis recurrence (OR 5.63).

NST techniques in high-risk surgical patients with acute cholecystitis have been compared by Mohan et al. in a large meta-analysis including 82 studies for a total of 15,131 patients (1223 treated with TP-GBD, 557 with EUS-GBD, and 13,351 with PGBD) [94]. The authors reported pooled technical and clinical successes for TP-GBD of 83% (95% confidence interval [CI]: 80.1–85.5) and 88.1% (95% CI: 83.6–91.4), respectively; for EUS-GBD, 95.3% (95% CI: 92.8–96.9) and 96.7% (95% CI: 94.0–98.2), respectively; and for PT-GBD, 98.7% (95% CI: 98.0–99.1) and 89.3% (95% CI: 86.6–91.5), respectively.

Current guidelines suggest, in centres experienced in the procedure, the use of EUS-GBD as the first choice in the drainage of AC unfit for surgery [8,78,95,96]. As said, the procedure should be performed within 24–48 h.

The endoscopic approach can indeed be considered a definitive treatment for patients unfit for surgery. Conversely, percutaneous drainage is the procedure of choice when expertise is not available for EUS-GBD in severely compromised patients with a surgical perspective, as a bridge before cholecystectomy. Delayed laparoscopic cholecystectomy in patients with PT-GBD is suggested in all those patients recovering from the conditions that previously discouraged surgical intervention after at least 6 weeks from the gallbladder drainage [97].

## 6. Conclusions

The endoscopic on-call system provides a trained endoscopist for urgent procedures, available 24/7.

Endoscopic procedures of the biliopancreatic tract, however, require specialized training that not all endoscopists, even those with much experience, may have.

An analysis of the literature shows that some diseases of the biliary tract require endoscopic treatment in an emergency, raising a challenging organization of trained personnel in the Gastroenterology Units.

**Figure 2:**
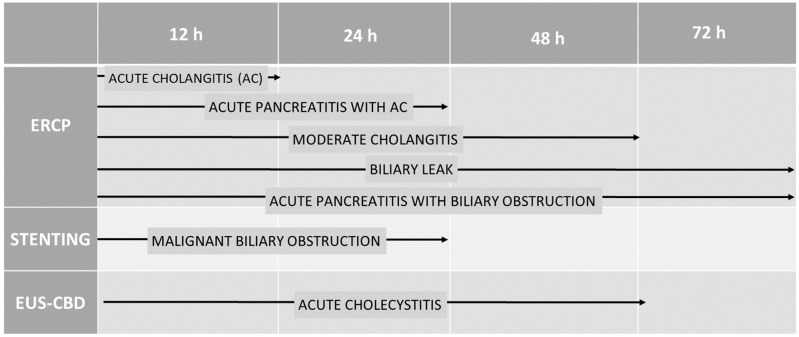
Performance time of procedures according to type and clinical condition.

Severe ACh is a disease of greater emergency, which, according to the ESGE guidelines, should be treated with ERCP within 12 h; in the case of ABP associated with cholangitis, the available studies show an extended operative window of up to 24 h. All other conditions evaluated could be delayed between 24 and 72 h (Figure 2).

Thus, it can be assumed that a full weekend can be waited for most procedures, whereas for severe cholangitis, with or without associated acute pancreatitis, a biliary endoscopist, available at least during daytime hours 7 days a week, should be provided.

The organization is more complex; in fact, it is not only necessary to consider the need for a specifically trained gastroenterologist—ERCP requires a dedicated team made by nursing staff and, in some hospitals, radiology technologists to manage the fluoroscopy equipment. Furthermore, most ECRPs are nowadays performed with anesthesia, thus requiring an additional nurse and an anesthesiologist. In summary, it is a procedure that requires the coordination of numerous professional figures and the use of advanced equipment.

Multiple studies have shown that ERCP performed after-hours or over the weekend is as safe as those performed during standard weekday hours [98,99,100]; thus, considering the guidelines and the clinical benefit of patients, the organization of the Gastroenterology Units should be reviewed.

In conclusion, every Gastroenterology Unit should provide at least a daytime availability for urgent biliary tract procedures 7 days a week for the management of severe ACh. Patients with ABP, AC unfit for surgery, and not responsive to medical therapy or BDLs can be treated over a longer period, allowing its scheduling on the first available day of the week.

## Figures and Tables

**Table 1:** Severity classification of Ach according to TG18/TG13 Guidelines [6,8].

Grade III (Severe)	Ach associated with dysfunction at least in one of the following: 1. Cardiovascular: hypotension requiring dopamine ≥ 5 Lg/kg per min, or any dose of norepinephrine; 2. Neurological: disturbance of consciousness; 3. Respiratory: PaO_2_/FiO_2_ ratio < 300; 4. Renal: oliguria, serum creatinine > 2.0 mg/dL; 5. Hepatic: PT-INR > 1.5; 6. Hematological: platelet count < 100,000/mm.
Grade II (Moderate)	Ach associated with any two of the following: 1. Abnormal WBC count (>12,000/mm^3^, <4000/mm^3^); 2. High fever (≥39 °C); 3. Age (≥75 years old); 4. Hyperbilirubinemia (total bilirubin ≥ 5 mg/dL); 5. Hypoalbuminemia (<STD × 0.7 ^a^).
Grade I (Mild)	Grade I Ach does not meet the criteria of “Grade III” or “Grade II” ACh at initial diagnosis

^a^ STD: lower limit of normal value.

**Table 3:** Main studies comparing early ERCP (at different timepoints) with conservative treatment for AP without cholangitis.

Author	Year	Patients (ERCP/Control)	Timing (h)	Mortality (%)	Morbidity (%)	Pancreatic Fluid Collection (%)	Organ Failure/Sepsis (%)
Folsch [53]	1997	126/112	<72	7.9/3.6	46/50.9	23/22.3	34.1/25.9
Oria [52]	2007	51/51	<72	5.9/2	37.3/29.4	11.8/9.8	25.5/19.6
Schepers [51]	2020	117/113	<24	6.8/8.8	28.5/44.2	14.5/15.9	18.8/15

Modified from Mukai et al. [49].

## Data Availability

No new data were created or analyzed in this study.

## Abbreviations

The following abbreviations are used in this manuscript:

ABP	Acute biliary pancreatitis
AP	Acute pancreatitis
AC	Acute cholecystitis
ACh	Acute cholangitis
ASGE	American Society of Gastrointestinal Endoscopy
BDL	Biliary duct leak
DPS	Double pig-tail stent
ESGE	European Society of Gastrointestinal Endoscopy
ERCP	Endoscopic retrograde cholangiopancreatography
EUS	Endoscopic ultrasound
EUS-BD	Endoscopic ultrasound-guided biliary drainage
EUS-GBD	EUS-guided gallbladder drainage
LAMS	Lumen-apposing metal stent
MBO	Malignant biliary obstruction
PTBD	Percutaneous transhepatic biliary drainage
PT-GBD	Percutaneous transhepatic gallbladder drainage
TP-GBD	Trans-papillary gallbladder drainage
SEMS	Self-expanding metal stent
WSES	World Society of Emergency Surgery
